# Calcineurin inhibitor monitoring in allogeneic hematopoietic cell transplantation: time to move beyond solid organ transplant paradigms

**DOI:** 10.3389/fimmu.2026.1940626

**Published:** 2026-09-22

**Authors:** Pedro Henrique Prata, Marc Labriffe, Pierre Marquet, Jean-Baptiste Woillard, Caroline Monchaud

**Affiliations:** 1 Department of Clinical Hematology and Cell Therapy, Centre Hospitalier Universitaire (CHU) de Limoges, Limoges, France; 2 Pharmacology & Transplantation, Université de Limoges, Limoges, France; 3 Department of Pharmacology, Toxicology and Pharmacovigilance, CHU de Limoges, Limoges, France

**Keywords:** allogeneic stem cell transplantation, AUC, calcineurin inhibitor (CNI), GvHD, MIPD, TDM

## Abstract

Calcineurin inhibitors, such as cyclosporine and tacrolimus, have been the backbone of graft-versus-host disease prophylaxis in allogeneic hematopoietic cell transplantation for more than four decades. Yet, the pharmacological framework governing their therapeutic drug monitoring has been significantly imported from solid organ transplantation. This critical narrative review argues that this extrapolation is scientifically unsound. The unique biological environment of allogeneic hematopoietic cell transplantation characterized by toxic conditioning regimens, profound hematologic changes, systemic inflammation, and an unparalleled drug interaction landscape creates pharmacokinetic and pharmacodynamic conditions for which solid organ transplant-derived targets are inadequate. We critically appraise the quality of evidence underpinning current therapeutic drug monitoring practices, examine the historical divide between cyclosporine and tacrolimus use, and propose a research agenda directed at developing hematopoietic transplant-specific precision immunosuppression strategies.

## 1 Introduction

The first successful allogeneic bone marrow transplantation, reported by Thomas et al. in 1957 (1), opened a therapeutic frontier that would transform the treatment of hematologic malignancies, bone marrow failure syndromes, and inherited disorders of hematopoiesis. In the early era of allogeneic transplantation, transplant-related mortality exceeded 50–70%, driven by three interdependent complications: graft rejection, severe acute graft-versus-host disease (GvHD), and opportunistic infections (2). These outcomes defined the central problem of allogeneic transplantation: how to achieve durable immune reconstitution while preventing the graft from killing the host.

The first immunosuppressive strategy to show efficacy came from animal models and used methotrexate (MTX) (3), based on its antiproliferative effects on rapidly dividing T cells. Subsequently, a randomized trial compared the efficacy of cyclosporine A against methotrexate, showing cyclosporine was an effective alternative, with less toxicity (4). However, it was the landmark randomized trial that established the combination of both drugs, cyclosporine and MTX, as the gold standard for GvHD prophylaxis (4, 5), a benchmark that remains today the reference against which all subsequent strategies are compared. This study demonstrated that combination prophylaxis significantly reduced the incidence of acute GvHD and improved survival compared with cyclosporine alone.

More than four decades later, calcineurin inhibitors (CNIs) continue to underpin GvHD prophylaxis across nearly all transplant platforms (matched sibling, matched unrelated, haploidentical, and cord blood) and across all conditioning intensities. Even the sharp expansion of post-transplant cyclophosphamide (PTCy)-based regimens in the last decade, which has transformed haploidentical and increasingly unrelated donor transplantation, has not displaced CNIs but rather incorporated them as essential partners in a multi-agent backbone (6, 7). The question is no longer whether to use CNIs, but how to use them optimally.

Still, there is a profound irony at the heart of modern transplant practice: despite this central and enduring role, the way we prescribe, monitor, and titrate CNIs in hematopoietic cell transplantation (HCT) is largely based on evidence generated in an entirely different clinical setting — solid organ transplantation. This critical narrative review challenges that paradigm, examines its scientific foundations, and argues that the time has come to develop HCT-specific pharmacological strategies.

## 2 The emergence of calcineurin inhibitors

### 2.1 Discovery of cyclosporine

The story of cyclosporine begins in 1969 with a soil sample collected in Norway subsequently characterized as containing the fungus *Tolypocladium inflatum*. The Belgian biologist Jean-François Borel, working at Sandoz Pharmaceuticals in Basel, identified in 1972 that the cyclic undecapeptide produced by this organism possessed selective immunosuppressive properties without the broad cytotoxicity of existing agents (8). His work demonstrated dose-dependent suppression of lymphocyte proliferation with relative sparing of other rapidly dividing cell populations — a selectivity that distinguished cyclosporine from all preceding immunosuppressants, especially antimetabolites as MTX.

The clinical translation was rapid. Roy Calne at Cambridge published the first results in renal transplant recipients in 1978 (9), reporting improvements in graft survival. Cyclosporine’s mechanism — the inhibition of calcineurin (10), a phosphatase required for nuclear factor of activated T cells (NFAT) dephosphorylation (11, 12) and subsequent interleukin-2 transcription (13) — was subsequently elucidated and provided a rational framework for immunosuppression targeting antigen-driven T-cell activation. The impact on solid organ transplantation was revolutionary: one-year kidney graft survival rates improved from approximately 50% to over 80% (9), changing the therapeutic landscape.

### 2.2 Development of tacrolimus

Tacrolimus (FK506) was isolated in 1984 from the soil bacterium *Streptomyces tsukubaensis* by researchers at Fujisawa Pharmaceutical Company in Japan (14). Despite sharing the same molecular target, the calcineurin-NFAT axis, tacrolimus operates through a structurally and mechanistically distinct pathway: it binds FKBP12 (FK-binding protein 12) (15), whereas cyclosporine binds cyclophilin A (16). The resulting complexes both inhibit calcineurin but with substantially different potencies; *in vitro* studies demonstrate that tacrolimus is approximately 10- to 100-fold more potent on a molar basis than cyclosporine (17).

Tacrolimus was first approved for clinical use in liver transplantation in the United States in 1994, following pivotal trials demonstrating superior rejection prevention compared with cyclosporine-based regimens (18–20). Its rapid adoption in solid organ transplantation over the subsequent decade was driven by this enhanced efficacy profile, and it progressively displaced cyclosporine as the preferred CNI in most solid organ transplant programs (21).

## 3 How calcineurin inhibitors became the backbone of GvHD prevention

The trajectory of GvHD prophylaxis spans five decades and reflects the progressive layering of immunosuppressive strategies over an increasingly complex transplant landscape. MTX alone represented the first era, followed by the pivotal demonstration that cyclosporine alone could prevent GvHD (4), and then the establishment of cyclosporine-MTX combination as the standard (5). The introduction of tacrolimus combined with MTX added a more potent CNI-based option (22), and the subsequent incorporation of mycophenolate mofetil (MMF) as an alternative to or complement of MTX further expanded the toolkit (23).

A second major pillar of GvHD prophylaxis, anti-thymocyte globulin (ATG) evolved alongside CNIs. Polyclonal rabbit-derived serums, chiefly anti-human thymocyte globulin (marketed as Thymoglobulin®) and ATG-Fresenius (anti-Jurkat T cell globulin, marketed as Grafalon®), act through a complementary mechanism: *in vivo* host and donor T-cell depletion, inhibition of inflammatory cell trafficking, and relative preservation of regulatory T cells. Their clinical role was established through a series of phase 3 randomized controlled trials conducted predominantly in the unrelated donor setting. The pivotal ATG-Fresenius Trial demonstrated that adding ATG-Fresenius to standard cyclosporine-methotrexate prophylaxis in the myeloablative conditioning regimen (MAC) setting significantly reduced both grade III–IV acute GvHD (11.7% versus 25.5%) and extensive chronic GvHD (12.2% versus 45%), without increasing relapse or compromising overall survival (24). In the matched sibling setting, ATG-Fresenius added MAC significantly reduced chronic GvHD and improved the composite endpoint of chronic GvHD-free and relapse-free survival, again without detriment to overall survival (25). Data favors a survival benefit for Thymoglobulin® in unrelated donor transplantation, MAC and reduced intensity conditioning (RIC) regimens, and demonstrated that the primary clinical benefit was a sustained reduction in the requirement for long-term systemic immunosuppression (26) and a lower risk for both acute and chronic GvHD without compromising disease control (27).

Importantly, ATG does not replace CNIs in these protocols; it is added to them. All pivotal ATG trials were conducted against a CNI-based prophylaxis backbone. ATG and CNIs function as pharmacologically complementary agents: ATG depletes alloreactive T cells pre- and peri-engraftment, while CNIs suppress ongoing calcineurin-mediated T cell activation during the weeks and months that follow. ATG itself exhibits marked pharmacokinetic variability driven by inter-patient differences in lymphocyte burden, which determines the consumption and clearance of active ATG (28). There is growing evidence that ATG exposure modulates the effective immunosuppressive environment in ways that could directly influence optimal CNI targets (29).

Another significant paradigm shift of the past two decades has been the emergence of PTCy (6, 7), originally developed for haploidentical transplantation and subsequently demonstrated to be effective across donor types including unrelated donors. The hypothesized mechanism of PTCy — selective elimination of alloreactive T cells at peak activation while sparing regulatory T cells and non-alloreactive lymphocytes — is very different from CNI-mediated suppression. Nonetheless, even within PTCy protocols, CNIs (originally tacrolimus) remain a cornerstone, combined with MMF to provide complementary immunosuppression during engraftment.

The persistence of CNIs across all these platforms is not historical inertia alone; it reflects the need for a reliable, titratable immunosuppressive anchor during the period of maximal alloimmune risk. Therefore, the quality of CNI therapeutic drug monitoring (TDM) in HCT is a matter of direct clinical consequence. Subtherapeutic exposure risks GvHD; supratherapeutic exposure risks nephrotoxicity, neurotoxicity, and transplant-associated thrombotic microangiopathy (30), or even the suppression of the graft-versus-leukemia effect (31, 32). The precision with which we navigate this narrow therapeutic window determines GvHD risk and both relapse and non-relapse mortality.

## 4 The HCT environment is not solid organ transplantation

The central argument of this review is that, because the biological context of allogeneic HCT differs fundamentally from solid organ transplantation limiting the direct transferability of CNI therapeutic targets between these settings. The major differences affecting CNI pharmacokinetics and the interpretation of therapeutic drug monitoring are summarized in **Figure 1**. The next section examines the most important sources of divergence.

**Figure 1:**
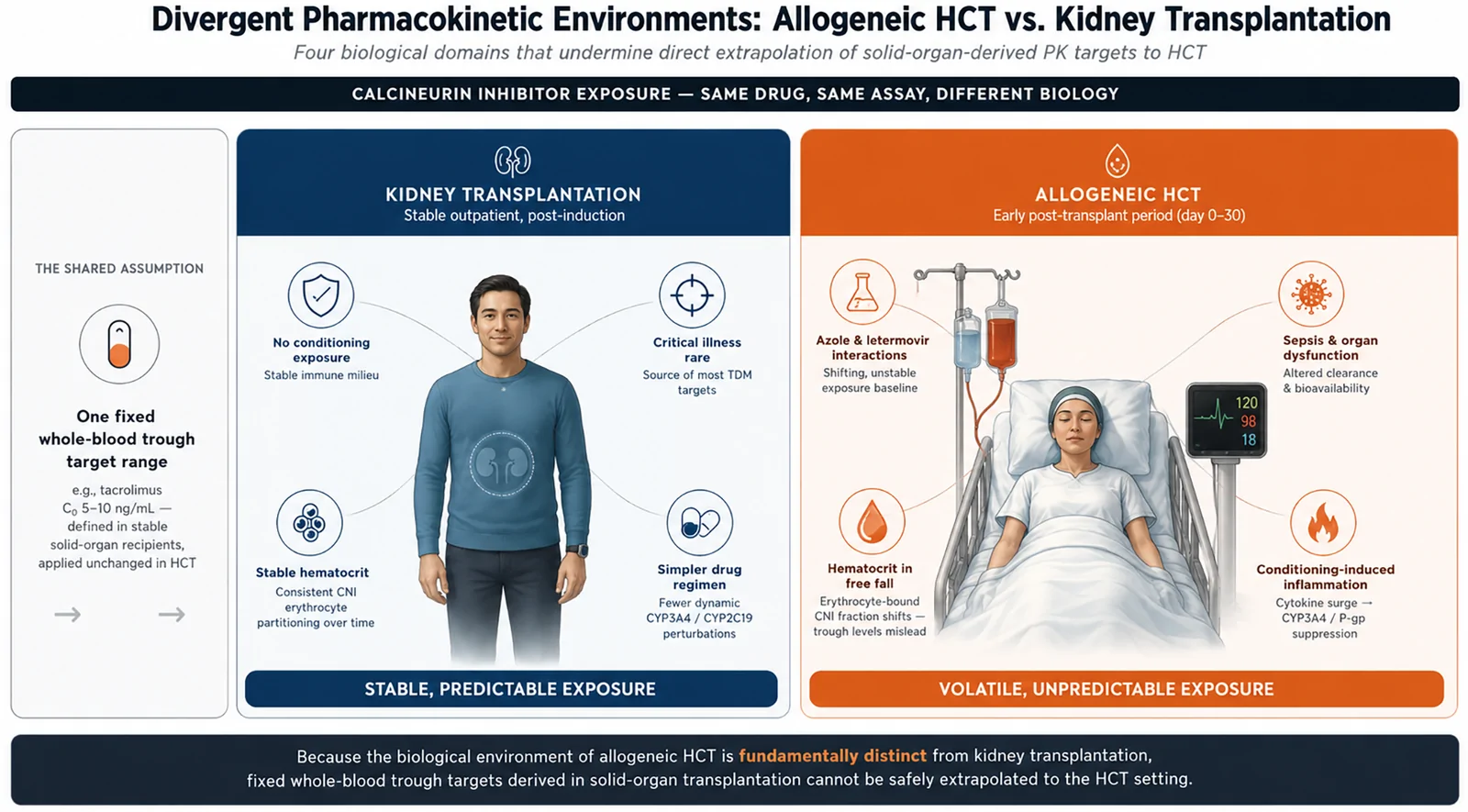
Why solid-organ CNI targets cannot be directly applied to allogeneic HCT. Although calcineurin inhibitors (CNIs) are monitored using the same whole-blood assays in both kidney transplantation and allogeneic hematopoietic cell transplantation (HCT), the biological determinants of drug exposure differ substantially. In kidney transplantation, therapeutic drug monitoring is typically performed in clinically stable recipients with preserved hematocrit, limited inflammatory perturbation, and relatively predictable pharmacokinetics. In contrast, early post-transplant HCT is characterized by conditioning-induced inflammation, profound fluctuations in hematocrit, critical illness, organ dysfunction, and frequent exposure to interacting medications such as azoles and letermovir. These factors alter CNI distribution, metabolism, and bioavailability, resulting in highly variable exposure despite similar measured trough concentrations. Consequently, therapeutic targets established in solid-organ transplantation cannot be assumed to be directly applicable to the HCT setting.

### 4.1 Conditioning-induced inflammation

Conditioning regimens generate an unparallelled systemic inflammatory state. Total body irradiation (TBI) and high-dose alkylating agents produce profound gastrointestinal epithelial barrier disruption, mucositis, and the liberation of pathogen and damage-associated molecular patterns (PAMP and DAMP, respectively) within 24–72 hours of administration (33). This results in the translocation of bacterial lipopolysaccharide and other microbial products into the systemic circulation, triggering the release of pro-inflammatory cytokines as TNF-α, IL-6, and IL-1β.

These conditioning-induced inflammatory changes have direct consequences for CNI pharmacokinetics. Cytokine-mediated suppression of CYP3A4 and P-glycoprotein expression alters both intestinal absorption and hepatic clearance of tacrolimus and cyclosporine (34). The inflammatory milieu also modulates protein binding, as both CNIs are protein-bound (to lipoproteins and in erythrocytes), and the acute-phase protein shifts and anemia that characterize the early post-transplant stage alter binding dynamics in ways that cannot be captured by whole-blood concentration monitoring. Besides, a kidney transplant recipient at any time after transplant is in an extremely different inflammatory state than an HCT recipient on day +5.

### 4.2 Profound hematologic changes

Arguably, the most underappreciated pharmacokinetic challenge in HCT is the rapid and dynamic change in hematocrit. Following conditioning, hematocrit may fall from close to normal to 20% within one to two weeks, before recovering progressively over the ensuing months as donor engraftment proceeds. This is clinically critical because both cyclosporine and tacrolimus exhibit extensive partitioning into erythrocytes, making whole-blood concentrations highly dependent on hematocrit. Cyclosporine distributes predominantly between erythrocytes (approximately 40–50%), plasma (30–40%), and leukocytes (10–20%) (35), whereas tacrolimus displays even greater erythrocyte binding, with the majority of circulating drug residing within red blood cells (21, 36).

The clinical implication is significant: can whole-blood trough concentrations be interpreted similarly in a patient whose hematocrit zigzags 10–20 percentage points over a single week? The answer is, probably, no. A given whole-blood CNI concentration in a patient with a hematocrit of 35% reflects a *substantially* different free plasma concentration than the same trough in a patient with a hematocrit of 18%. Because CNIs extensively partition into erythrocytes, a lower hematocrit reduces red blood cell binding and results in a greater proportion of the drug remaining in plasma. Consequently, for the same whole-blood concentration, patients with low hematocrit may have substantially higher pharmacologically active plasma concentrations and tissue exposure. Standard TDM, which reports whole-blood concentrations without hematocrit correction, may misclassify exposure status during the critical early post-transplant period*, i.e.*, precisely when accurate dosing matters most.

### 4.3 Sepsis and critical illness

Sepsis during the neutropenic phase of HCT is common and frequently complicated by hypoalbuminemia, hepatic dysfunction, and reduction in intestinal absorptive capacity (mucositis). Each of these perturbations affects CNI pharmacokinetics. Hepatic clearance, the main elimination pathway for both agents, is reduced in the setting of hepatic dysfunction or sinusoidal obstruction syndrome. Diarrhea decreases oral bioavailability, which is already highly variable for both CNIs.

In stable renal transplant recipients (the population from which most TDM targets are derived) such episodes of critical illness are uncommon. In HCT, they are very frequent at the early post-transplant course. The pharmacokinetic models and target ranges validated in a population of medically stable kidney recipients sitting in a transplant outpatient clinic bear limited relevance to a febrile, inflamed, neutropenic HCT recipient on broad-spectrum antibiotics in hospital.

### 4.4 Drug interactions: HCT as a pharmacological maze

Allogeneic HCT creates a drug interaction environment of exceptional complexity, unmatched in any other area of medicine. The polypharmacy burden derives from simultaneous requirements for antifungal and antiviral prophylaxis, antibacterial agents, antiemetics, analgesics, mucositis management, and conditioning-related supportive care, all administered to patients whose CYP3A4 and P-glycoprotein activity are already perturbed by inflammation and conditioning.

Azole antifungal agents are potent CNI interactants in clinical practice. Voriconazole and posaconazole are strong CYP3A4 and CYP2C19 inhibitors, increasing manyfold tacrolimus and cyclosporine exposure without dose adjustment (37, 38). The magnitude of the interaction is highly variable between individuals and fluctuates as antifungal therapy is started, modified in dose, or discontinued — creating an ever-shifting baseline against which trough concentrations must be interpreted.

Letermovir, now widely used for CMV prophylaxis post-HCT (39, 40), inhibits the CYP3A4/P-glycoprotein pathway and leads to significant increases in CNI exposure that are not always dose-adjusted appropriately in clinical practice. A further layer of complexity arises from the voriconazole – letermovir interaction: letermovir is a CYP2C19 inducer, whereas voriconazole is a CYP3A4 inhibitor. In patients receiving the triple combination of a CNI, voriconazole, and letermovir, these opposing effects on drug metabolism create a highly unpredictable net impact on CNI exposure, underscoring the critical importance of intensive TDM monitoring in this setting. Likely, the cumulative pharmacokinetic impact of this polypharmacy – which shifts dynamically as individual drugs are added, removed, or dose-adjusted — cannot be adequately managed by trough monitoring alone.

## 5 Cyclosporine versus tacrolimus: the great divide

### 5.1 The use of cyclosporine

The historical divergence in transplant practices concerning CNI choice represents one of the most striking geographical differences in evidence-based medicine. The use of cyclosporine is reinforced by a body of observational data demonstrating adequate outcomes with cyclosporine-based prophylaxis, by established institutional protocols with well-characterized cyclosporine targets, and by the practical advantages of intravenous formulation availability and lower cost.

There was also, implicitly, an awareness that the evidence for tacrolimus’s superiority in solid organ transplantation, primarily driven by rejection prevention, might not directly translate to the GvHD prevention context, where the immunological target is different.

### 5.2 The use of tacrolimus

North American adoption of tacrolimus was driven principally by the two landmark randomized trials, comparing tacrolimus-MTX with cyclosporine-MTX after HLA-identical, related and unrelated allogenic HCT (20, 22). Both studies demonstrated a reduction in grade II–IV acute GvHD with tacrolimus-MTX compared with cyclosporine-MTX. These results, combined with the parallel evidence from liver and kidney transplantation, drove progressive North American adoption of tacrolimus over the subsequent decade.

The adoption was not universal or immediate, but by the early 2010s, tacrolimus had become the dominant CNI in most North American academic transplant centers, while many European programs retained cyclosporine as the default, creating a persistent and largely unresolved difference.

### 5.3 Why solid organ transplantation abandoned cyclosporine and why this does not resolve the HCT question

In solid organ transplantation, the replacement of cyclosporine by tacrolimus was driven by robust evidence demonstrating superior prevention of acute and chronic rejection, improved long-term graft survival, and a more favorable drug interaction profile (18, 19, 34, 41). This evidence was generated in populations and contexts where prevention of allograft rejection is the primary immunological objective and where the absence of a beneficial graft-versus-tumor effect means that over-immunosuppression carries no therapeutic benefit, only toxicity risk.

The HCT setting is different. The immunological objective is not simply preventing rejection (indeed, the graft must ‘take’ and restore hematopoiesis) but rather calibrating the graft-versus-host/graft-versus-leukemia (GvH/GvL) balance: enough immune activity to prevent relapse, insufficient to cause severe GvHD. CNIs suppress both GvHD and GvL through their T-cell inhibitory effects, and the optimal exposure window in HCT must therefore reflect this dual requirement and may vary according to the intensity and timing of the overall immunosuppressive strategy. This consideration is entirely absent in solid organ transplantation (31, 32, 42–44).

This fundamental difference in the immunological objective constitutes the strongest argument against extrapolating solid organ transplant evidence to HCT. Superiority in kidney transplantation does not imply superiority in HCT, because the criteria for success differ in a clinically critical way.

### 5.4 Is tacrolimus truly better in HCT?

The question of whether tacrolimus is superior to cyclosporine in HCT remains unsettled. The pivotal trials demonstrated reduced acute GvHD with tacrolimus-MTX, but neither trial demonstrated a significant survival benefit (20, 22, 45). The reduction in acute GvHD incidence was not accompanied by a consistent reduction in relapse or improvement in non-relapse mortality, leaving overall survival essentially equivalent between arms.

Subsequent analyses have raised the possibility that tacrolimus’s greater immunosuppressive potency, while reducing GvHD, might also more profoundly suppress GvL, potentially offsetting its acute GvHD benefit in certain disease contexts (31, 32). Additionally, tacrolimus allegedly carries a higher rate of treatment-associated thrombotic microangiopathy and, at supratherapeutic exposures, significant neurotoxicity. Such complications frequencies in the HCT setting may differ from solid organ transplantation due to the additional stressors of conditioning (30). Tacrolimus and cyclosporine exposures are also associated with dose-dependent acute kidney injury related to calcineurin inhibitor nephrotoxicity (46), particularly in the context of concomitant nephrotoxic therapies used after HCT (47). In addition, it increases the risk of post-transplant diabetes mellitus, primarily through β-cell dysfunction and impaired insulin secretion, with risk rising at higher systemic exposures (48).

## 6 Therapeutic drug monitoring: how much data comes from HCT?

A critical examination of the evidence base for CNI TDM in HCT reveals a striking paradox: despite decades of practice, most concepts in CNI TDM were developed and validated in solid organ transplantation.

The evidence specifically evaluating CNI TDM in the HCT context is notable for its limitations. Studies of cyclosporine TDM in HCT are predominantly retrospective and monocentric, with highly heterogeneous target ranges, conditioning platforms, and GvHD prophylaxis backbones (49). They address a range of endpoints, including acute GvHD, chronic GvHD, relapse, and nephrotoxicity, but without adequate power.

For tacrolimus, the pivotal trials establishing its use in HCT defined efficacy at population level but provided no exposure-response data beyond broad observations about trough concentrations and adverse events (20, 22). The exposure-response literature for tacrolimus in HCT remains limited to retrospective and single-center series (42). Therapeutic windows vary substantially between institutions: some programs target troughs of 5–10 ng/mL, others 8–12 ng/mL or 10–20 ng/mL, with little prospective evidence to differentiate between these targets. Importantly, this heterogeneity may not simply reflect a lack of standardization. The optimal CNI exposure may depend on the overall transplantation and immunosuppressive context. CNI initiation may occur before conditioning, around transplantation, or after transplantation depending on the GvHD prophylaxis platform, and CNIs may be combined with MTX, MMF, PTCy, ATG, or other agents with substantially different immunosuppressive effects. Consequently, an identical tacrolimus or cyclosporine concentration may not necessarily represent the same net degree of immunosuppression across these settings. Moreover, the optimal exposure may itself vary over time, as the competing risks of GvHD, relapse, infection, and drug-related toxicity evolve after transplantation. Thus, the objective should not necessarily be to establish a single universal therapeutic window, nor to accept empirically defined center-specific ranges, but rather to determine whether exposure targets should be adapted to the GvHD prophylaxis platform, timing of CNI initiation, post-transplant period, and relevant patient and transplant characteristics.

No randomized controlled trial has prospectively compared different tacrolimus exposure targets in HCT against clinically meaningful endpoints. AUC-guided dosing is the approach increasingly advocated in kidney transplantation as more predictive of outcomes than trough monitoring alone (50). No prospective validation exists in the HCT setting.

One of the few studies specifically designed to address cyclosporine pharmacokinetics in the HCT setting showed that interdose AUC can be reliably estimated using a limited sampling strategy, thereby providing a practical approach to AUC-based cyclosporine exposure assessment without the need for full pharmacokinetic profiling (51). However, this approach has not been implemented in routine hematology practice, and no validated interdose AUC target is currently available to guide cyclosporine dose adjustment in this setting. This should be distinguished from tacrolimus monitoring, for which the average trough concentration exposure during the first three months of therapy has been reported to be independently associated with overall survival (52).

Together, these contributions represent exceptions that underscore the rule: the vast majority of CNI TDM evidence informing HCT practice derive from populations, models, and targets developed in very different settings.

## 7 Beyond trough concentrations

### 7.1 The limitations of trough monitoring

Trough concentration (C0) monitoring (the measurement of the whole-blood CNI level immediately before the next dose, ideally 12h (if BID) or 24h (if QD) after the previous dose) remains the dominant TDM strategy in both solid organ transplantation and HCT despite the limitations we have discussed in the previous sections. Its persistence reflects logistical simplicity: a single pre-dose blood sample is required, and sampling time is standardized within routine whole-blood CNI TDM workflows. These advantages are primarily logistical rather than analytical. However, it is now well established in solid organ transplantation that CNI C0 is an imperfect surrogate for the area under the concentration-time curve (AUC), the metric that best estimates total drug exposure, because the concentration-time profile shows marked intra- and inter-individual variability.

The poor correlation between C0 and AUC is not a theoretical concern. It is quantified and largely reported. In solid organ transplantation, the r² between tacrolimus C0 and AUC(0–12h) ranges widely across studies, from as low as 0.50 to as high as 0.85 depending on the population, time post-transplant, and formulation. This range, at its lower end, indicates that trough concentration explains less than half the variability in true drug exposure (53, 54). For cyclosporine, the problem is even more pronounced: in HCT-specific cohorts, C0 shows poor correlation with AUC(0–12h) in both intravenous and oral administration (51).

In HCT, the limitations of trough monitoring are further magnified by the dynamic pharmacokinetic conditions described above. A trough concentration measured in the context of a falling hematocrit, active azole antifungal use, and post-conditioning inflammation reflects a composite of multiple confounding variables that are entirely absent in the stable outpatient kidney transplant recipient. The biological meaning of a therapeutic trough in this context is, at least, uncertain (50, 53, 54).

### 7.2 AUC-guided dosing

AUC-guided dosing, in which total drug exposure over a dosing interval is used to individualize dose adjustment, offers a more complete characterization of CNI exposure than trough monitoring alone. In practice, this approach does not require a distinct analytical infrastructure when based on whole-blood CNI concentrations, but it does require pharmacokinetically informative sampling and access to validated tools, typically model-informed precision dosing (MIPD) or Bayesian platforms, to estimate individual AUC and translate it into dose adjustment. The main barriers to implementation are therefore not primarily analytical when whole-blood concentrations are used, but rather operational and pharmacometric, including the need for appropriately timed samples, validated population pharmacokinetic or Bayesian models, and accessible dose-adjustment tools.

In kidney transplantation, early cyclosporine exposure estimated by sparse-sampling AUC has been shown to be associated with acute rejection and cyclosporine nephrotoxicity more accurately than C0, providing a pharmacokinetic rationale for AUC-guided monitoring (55). For tacrolimus, AUC-based approaches are gaining traction in kidney transplantation, supported by population pharmacokinetic models that allow AUC estimation from sparse sampling strategies (50). Most recently, data support AUC-guided approaches over C0-based tacrolimus monitoring in kidney transplantation (56).

Furthermore, for tacrolimus use in HCT, higher systemic exposure appears to be associated with inferior overall survival, suggesting the existence of HCT-specific upper exposure thresholds that differ from those established in solid organ transplantation (52). This observation likely reflects the unique requirement to balance GvHD prevention with preservation of graft-versus-leukemia activity.

### 7.3 Population pharmacokinetics and Bayesian forecasting

Bayesian AUC estimation from a limited sampling strategy is both feasible and accurate for cyclosporine in adult HCT patients (51). It represents a promising framework for individualized CNI dosing in HCT. In this approach, a prior population pharmacokinetic model is updated with individual patient concentration observations to generate patient-specific pharmacokinetic parameter estimates and dose recommendations targeting a predefined exposure goal (41). The approach can leverage sparse sampling to generate exposure estimates when the underlying population model captures the relevant pharmacokinetic variability.

In the HCT setting, three independently developed Bayesian estimators performed with comparable predictive accuracy in a cyclosporine cohort, and a limited sampling strategy of two to three time points sufficed to estimate AUC reliably (51). For tacrolimus, a population PK model identified age as a significant covariate of clearance. This covariate is absent from renal transplant-derived models and it could have direct clinical implications for initial dosing in the HCT setting (52). Building and prospectively validating HCT-specific population PK models, incorporating hematocrit dynamics, conditioning intensity, azole exposure, and letermovir co-administration, represents a high-priority and feasible research objective.

### 7.4 Microsampling and future directions in TDM

Emerging microsampling technologies, particularly volumetric absorptive microsampling systems (VAMS), offer the possibility of decentralized, patient-performed sampling, enabling more frequent and pharmacokinetically informative monitoring without additional venous access requirements (57–60). These approaches are particularly attractive in the HCT setting, where patients undergoing outpatient post-transplant monitoring face high burdens of clinic attendance and venous sampling. Integration of microsampling with Bayesian forecasting platforms could enable adaptive, real-time dose optimization in the ambulatory post-transplant phase. This aim remains aspirational but is technically achievable with current technology.

### 7.5 Pharmacogenetics

Pharmacogenetic variability may contribute to the substantial interindividual variability in CNI exposure after allogeneic HCT. Both tacrolimus and cyclosporine undergo metabolism through CYP3A enzymes and are substrates of P-glycoprotein, encoded by ABCB1, providing a biological rationale for investigating variants in CYP3A4, CYP3A5, and ABCB1. However, the strength and clinical relevance of these associations differ substantially between the two CNIs and remain incompletely characterized in the HCT setting (61).

For tacrolimus, CYP3A5 is the best-established pharmacogenetic determinant. Carriers of at least one functional CYP3A51 allele (CYP3A5 expressers) have higher tacrolimus clearance and lower dose-adjusted concentrations than non-expressers, resulting in higher dose requirements (62). This relationship has also been demonstrated specifically in allogeneic HCT: in a prospective study of patients receiving oral tacrolimus, CYP3A51 expression was associated with lower dose-normalized concentrations and a longer time to achieve therapeutic exposure, whereas no independent effect of the investigated CYP3A4 or ABCB1 variants was demonstrated (62). Although genotype-guided tacrolimus dosing is supported by pharmacogenetic guidelines in solid organ transplantation, whether this strategy improves clinical outcomes in allogeneic HCT remains to be prospectively established.

For cyclosporine, the pharmacogenetic evidence is less established. A recent study in allogeneic HCT reported higher trough concentrations in carriers of CYP3A41B and CYP3A53 variants, suggesting that CYP3A genetic variability may contribute to cyclosporine exposure in this setting (63). However, previous studies, predominantly conducted in solid organ transplantation, have reported heterogeneous associations between CYP3A4, CYP3A5, and ABCB1 variants and cyclosporine pharmacokinetics (64, 65). Similarly, although ABCB1 is mechanistically relevant to the disposition of both CNIs, associations between common ABCB1 variants and systemic exposure or clinical outcomes have been inconsistent. Thus, pharmacogenetics represents a promising component of individualized CNI dosing in HCT, but at present CYP3A5-guided tacrolimus dosing has a substantially stronger evidence base than pharmacogenetic personalization of cyclosporine or ABCB1-guided dosing.

## 8 Future directions

There is a clear and substantial gap between the complexity of the HCT pharmacological environment and the evidence base informing CNI management in this setting.

Several registered and recently completed clinical studies address components of this research agenda. NCT04645667 prospectively characterized tacrolimus pharmacokinetic variability in adult allogeneic HCT recipients, incorporating intensive concentration–time sampling, pharmacogenetic information, pharmacodynamic biomarkers, and clinical outcomes, with the objective of supporting population PK/PD modeling and precision dosing. NCT06080490 evaluated tacrolimus exposure and transplant-related outcomes in pediatric HCT recipients, providing complementary evidence in a population in which CNI pharmacokinetics may differ substantially from adults. More recently, NCT07695571 was registered to evaluate a model-informed precision dosing strategy for cyclosporine in HCT recipients, including the development of individualized dosing models. Together, these studies illustrate an emerging shift toward HCT-specific CNI pharmacology and precision dosing. However, prospective multicenter studies specifically designed to establish and externally validate exposure targets against both efficacy and toxicity outcomes remain lacking. Closing this gap requires a coordinated research program focused specifically on the HCT context. **Box 1** summarizes key research priorities.

Box 1 **Research priorities for calcineurin inhibitor optimization in allogeneic HCT**

1. Prospective regional multicenter PK/PD studies linking CNI exposure to efficacy and toxicity outcomes

2. Development and validation of Bayesian estimators of the AUC of CNIs

3. Evaluation of pharmacogenetic-guided CNI dosing, particularly CYP3A5-guided tacrolimus initiation

4. Implementation of exposure–efficacy studies linking AUC to acute and chronic GvHD cumulative incidence, relapse, and survival

5. Implementation of exposure–toxicity studies (nephrotoxicity, neurotoxicity, thrombotic microangiopathy)

6. Derivation and external validation of protocol-, population-, and time-specific exposure targets, accounting for conditioning regimen, GvHD prophylaxis platform, timing of CNI initiation, graft source, donor type, and post-transplant period.

7. Implementation of randomized controlled studies evaluating the interest of MIPD

8. Evaluation of microsampling (VAMS) for therapeutic drug monitoring

9. Development and deployment of digital adherence measurement tools and remote TDM in the outpatient post-transplant period

The development of HCT-specific CNI management strategies will require collaboration across multiple disciplines: transplant physicians, clinical pharmacologists, pharmacokineticians, and biostatisticians.

Importantly, generating HCT-specific evidence does not necessarily require a single large multinational trial aimed at establishing a universal therapeutic range. Given the substantial geographical variation in CNI use, GvHD prophylaxis strategies, conditioning regimens, supportive care, and patient characteristics, a single target may itself be unrealistic. A more pragmatic strategy would consist of prospective regional multicenter PK/PD studies and harmonized observational cohorts linking CNI exposure to efficacy and toxicity endpoints, followed by external validation across independent populations. Such studies should prospectively capture the major determinants of CNI exposure and response, including concomitant medications, hematocrit, conditioning and prophylaxis regimens, and pharmacogenetic background. This framework could identify both broadly reproducible exposure–response relationships and situations in which population- or protocol-specific targets are warranted.

Finally, prospective studies should determine whether incorporation of pharmacogenetic information, particularly CYP3A5 genotype for tacrolimus, into HCT-specific dosing algorithms improves early target attainment and, ultimately, clinically relevant outcomes (66, 67).

## 9 Conclusion

CNIs have been the cornerstone of GvHD prophylaxis for more than four decades across all transplant platforms. The evidence reviewed here demonstrates that current CNI TDM in HCT relies on data derived chiefly in solid organ transplantation, *i.e.*, a fundamentally different biological setting characterized by distinct immunological objectives, stable hematological parameters, limited drug interaction complexity, and the absence of conditioning-induced inflammatory perturbation. The extrapolation of TDM targets, sampling strategies, and population pharmacokinetic models is not a minor translational shortcut; it is a significant source of pharmacological uncertainty with perhaps direct implications for patient outcomes. The next frontier is not the discovery of new immunosuppressive drugs, but the development of HCT-specific precision immunosuppression strategies, grounded in HCT-derived pharmacokinetic and pharmacodynamic data and capable of maximizing efficacy while minimizing toxicity in this unique physiopathological environment.
